# A Case of Hemophagocytic Lymphohistiocytosis Secondary to Disseminated Histoplasmosis

**DOI:** 10.1155/2020/6901514

**Published:** 2020-08-20

**Authors:** Ivan Columbus-Morales, Lucas Maahs, Sanam Husain, Stuart C. Gordon, Kedar V. Inamdar, Humberto C. Gonzalez

**Affiliations:** ^1^Department of Internal Medicine, Henry Ford Hospital, Detroit, MI, USA; ^2^Department of Pathology, Henry Ford Hospital, Detroit, MI, USA; ^3^Wayne State University, School of Medicine, Detroit, MI, USA; ^4^Department of Gastroenterology, Henry Ford Hospital, Detroit, MI, USA

## Abstract

Hemophagocytic lymphohistiocytosis (HLH) is a rare condition characterized by a pathologic immune dysregulation resulting in extreme inflammation. Clinical manifestations are varied but can include severe multiorgan failure and death. HLH has been associated with malignancies, autoimmune diseases, and infections, such as histoplasmosis. Histoplasmosis commonly has subclinical manifestations but can also present in its disseminated form. We present the case of an immunocompromised patient with worsening liver function caused by hepatic histoplasmosis that later triggered HLH with severe multiorgan dysfunction.

## 1. Introduction

Hemophagocytic lymphohistiocytosis (HLH) is a rare clinical syndrome characterized by fever, hepatosplenomegaly, cytopenias, presence of activated macrophages in hematopoietic organs, and progressive multiple organ failure [1]. The secondary form of HLH is often triggered by infections, malignancies, and autoimmune diseases [2]. Disseminated histoplasmosis (DH) has been described as a potential trigger, most commonly in immunocompromised hosts. The pathogenetic mechanisms behind HLH are not well understood but involve cytotoxic lymphocyte dysfunction leading to an exaggerated inflammatory response, which ultimately causes tissue damage and progressive multiple organ failure [3]. The liver, spleen, and lungs are the most frequently affected organs, but HLH can potentially affect all tissues in the human body. Patients with HLH are often severely ill making the establishment of the diagnosis challenging.

## 2. Case Presentation

A 42-year-old female presented to the emergency department with a 2-week history of jaundice, intermittent right upper quadrant abdominal pain, and fevers. Her past medical history was significant for ankylosing spondylitis for which she was on chronic immunosuppression with prednisone, methotrexate, and infliximab. On admission, she had raised liver biochemistries (aspartate aminotransferase 538 unit/L, alanine aminotransferase 132 unit/L, alkaline phosphatase 623 unit/L, total bilirubin 6.9 mg/dL, and direct bilirubin 6.2 mg/dL). The patient had no known history of liver disease, and her liver function tests were normal in the recent past. Additionally, her international normalized ratio was prolonged at 2.1, and platelets were 149,000 k/uL. Other laboratory values included ferritin (20,308 ng/dL) and triglycerides (1409 mg/dL) as well as low levels of fibrinogen (105 mg/dL). Soluble interleukin-2 receptor was positive. The peripheral smear showed toxic granulocytes and bandemia. Blood cultures, viral hepatitis panel, autoimmune panel, ceruloplasmin, and cytomegalovirus DNA were negative. A right upper quadrant ultrasonography revealed cholelithiasis, gallbladder wall thickening, and splenomegaly of 14 cm. A computed tomography scan of the abdomen confirmed the ultrasound findings and showed no evidence of intra- or extrahepatic biliary dilatation. A magnetic resonance cholangiopancreatography revealed severe gallbladder wall edema and a mild amount of fluid between it and the duodenum. She was initially started on broad-spectrum antibiotics to treat suspected acute cholangitis.

The patient clinically deteriorated with worsening mentation, persistent fever and was in need of vasopressor support and mechanical ventilation. The hospital course was further complicated by a tonic-clonic seizure and anuric acute kidney failure requiring renal replacement therapy. Given the clinical decline and uncertainty of the source of sepsis, antimicrobial coverage was broadened to include amphotericin B and acyclovir; a liver biopsy was performed.

**Figure 1:**
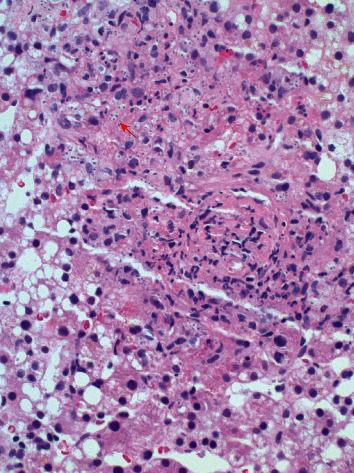
Hematoxylin and eosin stain. Liver biopsy showing noncaseating granulomas and intracellular fungal organisms.

**Figure 2:**
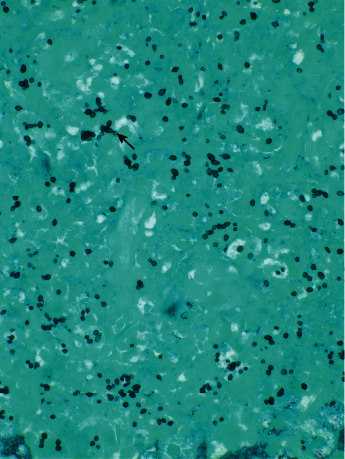
Grocott-Gomori methenamine silver stain: intracellular budding yeast formation.

The liver biopsy demonstrated marked granulomatous inflammation with numerous intracellular organisms that were morphologically compatible with *Histoplasma* (Figures 1 and 2).

**Figure 3:**
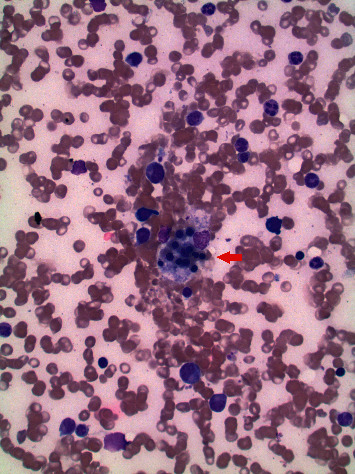
Leishman stain: hemophagocytosis in the bone marrow aspirate smear. The photomicrograph depicts a macrophage in the center of the smear ingesting nucleated red cell precursors.

**Figure 4:**
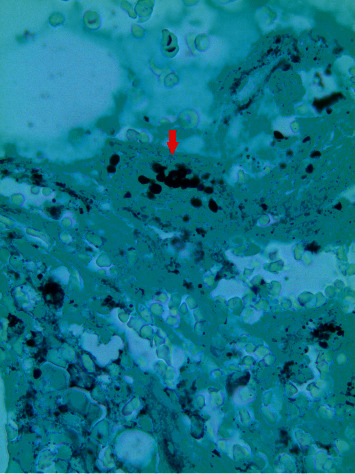
Grocott-Gomori methenamine silver stain: histoplasma organisms noted in bone marrow core biopsy showing yeast forms consistent with histoplasma species.

Serum histoplasma antigen was also positive. A bone marrow biopsy was obtained revealing hemophagocytic cells (Figures 3 and 4), but negative for any lymphoproliferative disorder. A Grocott-Gomori methenamine silver stain of the bone marrow was positive for fungal yeast forms (Figure 4).

Since the patient met diagnostic criteria for HLH, she was started on dexamethasone and etoposide. The patient received a total of 14 days of amphotericin B and then was transitioned to itraconazole. She steadily recovered, resolving the renal failure, allowing to discharge home. On follow-up, her blood counts and liver function tests had returned to normal, and her ferritin showed a marked improvement, 462 ng/mL and 195 ng/mL at 1 and 3 months, respectively. The histoplasma serum antigen became negative 1 month after and remained negative subsequently. Itraconazole had to be switched to posaconazole given gastrointestinal intolerance. Similar complaints persisted, and the patient was transitioned to isavuconazole. She remains off any immunosuppressants for ankylosing spondylitis.

## 3. Discussion

The initial clinical presentation in this case was suggestive of acute cholangitis; however, the history of chronic immunosuppression, cholestatic biochemical profile, poor clinical response to antibiotic therapy, and additional findings such as extreme hyperferritinemia, critically helped to establish the diagnosis of HLH secondary to DH. Previous case reports of HLH secondary to DH have been mostly associated to HIV/AIDS and after a solid organ transplant [4]. Patients with HIV can trigger HLH as consequence of acute HIV infection, initiation of antiretroviral therapy, immune reconstitution syndrome, or the presence of an opportunistic infection [5, 6]. Our patient, as well as other individuals with rheumatologic disorders (on immunosuppressant therapies), tend to have an opportunistic infection as the main trigger mechanism. Antitumor necrosis factor therapy (medication commonly used in autoimmune diseases) inhibits granuloma formation and increases the risk of systemic infections such as histoplasmosis [7].

Histoplasmosis, the most common endemic mycosis in the United States, is usually transmitted after inhalation of the organism, which is present in soils contaminated by bird or bat droppings [8]. Upon additional questioning, the patient acknowledged a history of raising chickens at home. DH is more common in the immunocompromised host and extreme of ages manifesting with high-grade fever, fatigue, weight loss, hepatosplenomegaly, and lymphadenopathy. Liver involvement presents with elevation of aspartate aminotransferase, alanine aminotransferase, and alkaline phosphatase, all of which were seen in this case [9]. Diagnostic tests include blood culture (gold standard), antigen detection, polymerase chain reaction assays, and direct microscopic examination of specimens, such as bronchial aspirates, bone marrow biopsy, or peripheral blood smear [10]. DH is treated with amphotericin B for severe infections and itraconazole for mild-to-moderate disease [11].

Despite the presence of a fungal infection as an explanation for extreme hyperferritinemia and a liver biopsy without evidence of hemophagocytes, the diagnosis of HLH was pursued. HLH presents as a febrile illness with multiorgan involvement including splenomegaly, cytopenias, coagulation abnormalities, extreme hyperferritinemia, hypertriglyceridemia, hemophagocytosis, and elevation of the soluble interleukin 2 receptor [1, 12]. Liver biochemistries are universally abnormal, and neurological manifestations such as ataxia, mental status changes and seizures are present in up to a third of the patients with HLH [13, 14]. Our patient met all of the above criteria and also had a bone marrow biopsy which is recommended early in the evaluation of patients meeting HLH 2004 diagnostic criteria [15]. Infection-related HLH cases should be treated aggressively with standard HLH protocols that include dexamethasone, etoposide, and cyclosporine [10, 14]. Mortality rates have been found to be high regardless of treatment modality in a previous case series [15].

If initial clinical recovery is achieved, recommendations include completing antifungal therapy for a year [16]. In this case, the patient had significant side effects associated to first- and second-line therapies requiring starting isavuconazole. There is minimal experience using this agent for histoplasmosis [17–19] as compared to other established antifungals.

This case confirms the challenges of diagnosing DH given the diverse clinical manifestations; however, persistent fever and a cholestatic biochemical profile should raise the index of suspicion in the right clinical setting. Awareness of HLH not only avoids missing a life-threatening condition but also prevents overdiagnosis that can lead to administration of corticosteroids and cytotoxic agents in critically ill individuals. Early recognition is crucial for a reasonable attempt at curative therapy, as treating the triggering event alone is usually not sufficient. Prevention of re-exposure and follow-up laboratory studies such as serial serum histoplasma antigen to monitor response to therapy is critical for prognosis, as mortality rates are exceeding high if relapse presents [20].

## Data Availability

No data were used to support this study.
